# Effects of bovine respiratory disease on the plasma metabolome of beef steers during the receiving period

**DOI:** 10.3389/fvets.2023.1239651

**Published:** 2023-08-04

**Authors:** Francisca O. Eichie, Godstime Taiwo, Modoluwamu Idowu, Taylor Sidney, Emily Treon, Deborah Ologunagba, Yarahy Leal, Ibukun M. Ogunade

**Affiliations:** Division of Animal Science and Nutritional Science, West Virginia University, Morgantown, WV, United States

**Keywords:** amino acids, bovine respiratory disease, metabolome, receiving period, beef cattle

## Abstract

The study aimed to investigate the impact of Bovine Respiratory Disease (BRD) on the metabolism of beef steers during a 35-d receiving period using plasma metabolomics. In this study, 77 newly weaned crossbred (Angus × Hereford) beef steers (BW = 206 ± 12 kg and age = 180 ± 17 days) were categorized into two groups: Healthy and Sick groups. The Sick group comprised beef steers diagnosed with BRD at any time during the 35-day period (*n* = 31), while the Healthy group did not show any signs of BRD (*n* = 46). Blood samples were collected from the coccygeal vessels on day 35, and plasma samples were subjected to targeted metabolomics analysis using Nuclear Magnetic Resonance spectroscopy. Data and statistical analyses, including biomarker and pathway enrichment analyses, were performed using Metaboanalyst 5.0. Results of the growth performance showed that sick steers had lower (*p* ≤ 0.05) ADG (1.44 vs. 1.64 kg/d) and higher (*p* = 0.01) feed:gain ratio (3.57 vs. 3.13) compared to healthy steers. A total of 50 metabolites were quantified. The partial least squares discriminant scores plot showed a slight separation between the two groups of steers, indicating some metabolic differences. Furthermore, the plasma concentrations of four metabolites (sarcosine, methionine, dimethyl sulfone, and L-histidine) were greater (*p* ≤ 0.05) in healthy steers compared to sick steers. Among these metabolites, sarcosine and methionine qualified as candidate biomarkers associated with BRD infection based on an area under the curve >0.70. Additionally, quantitative enrichment analysis revealed that cysteine and methionine metabolism was enriched in healthy steers compared to sick steers. This suggests that these metabolic pathways may play a role in the response to BRD infection. The findings of this study highlight the altered plasma metabolome in steers with BRD during the receiving period. Understanding these metabolic changes can contribute to the development of effective management strategies and nutritional interventions to mitigate the negative impact of BRD on beef cattle health and immune function.

## Introduction

Bovine respiratory disease (BRD) is a significant endemic disease that has had a considerable impact on morbidity and mortality rates in feedlots in the United States (1, 2). The complexity of BRD in the feedlot industry arises from various environmental and physiological stress factors that cattle encounter during the critical receiving period (3, 4). These stressors include weaning, dietary changes, mixing of unfamiliar animals, transportation, and exposure to pathogenic agents (4). During the feedlot receiving period, cattle often experience reduced DMI, leading to impaired nutrient metabolism and compromised immune function (5, 6). The combination of these factors creates an environment conducive to the development and progression of BRD.

Vaccination and antibiotic treatments have been integral in the prevention and mitigation of BRD in beef cattle (7–9). These interventions have proven effective in reducing the severity of BRD (10, 11). However, it is essential to address the public concern surrounding the use of antibiotics in food animals, as this issue is likely to persist (1, 13). Therefore, in addition to vaccination and antibiotic treatments, it becomes necessary to implement other management strategies to mitigate the negative impact of BRD on beef cattle health and immune function (14, 15). In recent times, the application of metabolomics has provided a blueprint for the comprehensive analysis of metabolites in biofluids, which has emerged as a powerful tool for understanding disease processes and identifying metabolic alterations associated with various conditions (16–18). By exploring the impact of BRD on overall metabolism, effective strategies for managing and implementing nutritional interventions can be utilized to mitigate the detrimental effects of BRD infection on the nutritional status and performance of beef cattle. Indeed, a recent study (19) demonstrated that dairy calves deliberately infected with BRD causal agents exhibited altered metabolism. However, the metabolism of beef cattle infected with BRD during the receiving period has not been fully described. Therefore, the objective of this study was to determine the effects of BRD infection on the plasma metabolome of beef steers during a 35-d receiving period. We hypothesized that BRD infection would induce alterations in the plasma metabolome of beef steers during this critical period.

## Materials and methods

### Animals, housing, and feeding

All animal care and use procedures adhered to the guidelines for the use of Animals in Agriculture Teaching and Research at West Virginia University (Protocol #2014-0194). Seventy-seven (77) newly weaned crossbred (Angus × Hereford) beef steers (BW = 204 ± 14 kg; age = 180 ± 17 days) were sourced from a single farm located about 150 miles away from the research station. Following initial vaccination at approximately 60 days of age, the beef steers received booster shots at approximately 7 d prior to weaning. This vaccination protocol involved two separate vaccinations, Pyramid 5 plus presponse sq. and Alpha-7/MB-1 (Boehringer Ingelheim Animal Health, Duluth, GA). Subsequently, the beef steers were transported to the university research farm (over a distance of approximately 150 miles). Upon arrival, the beef steers were weighed and processed. Processing procedures included the application of ear tags for unique radiofrequency identification purposes, as well as the administration of de-wormers (Valbazen, Zoetis Inc., Kalamazoo, MI). After being grouped according to their body weight on day 0, the steers were stratified into four pens, each pen housing 20 steers. This stratification was implemented to ensure that the starting body weights within each pen were similar. Each pen measured 14.6 by 46.9 m^2^ and was equipped with two GrowSafe intake nodes (GrowSafe Systems Ltd., Airdrie, Alberta, Canada) for individual feed intake measurement. The beef steers were fed a total mixed ration (TMR; Supplementary Table S1) formulated according to the recommendations for growing beef cattle (20) for a duration of 35 days.

### Intake and BW measurement

Individual feed intake was measured using GrowSafe intake nodes (GrowSafe Systems Ltd., Airdrie, Alberta, Canada). A 24-h intake period was defined from 0900 h to 0800 h the following day. Daily samples of the TMR were collected. These samples were weighed and subjected to oven drying at 55°C for a duration of 72 h to determine the dry matter (DM) content. The samples were then ground using a Wiley mill (Arthur H. Thomas Co., Philadelphia, PA) to pass through a 2-mm sieve. These ground subsamples were sent to Dairy One Forage Laboratory (Ithaca, NY) for chemical composition analysis. The BW of the beef steers were recorded prior to the morning feeding on d 0 and 35 of the experiment to calculate the average daily gain (ADG) of the beef steers.

### BRD morbidity and blood collection

The beef steers were subjected to daily visual examinations, and appropriate measures were taken to address sickness when necessary. To qualify for treatment, an animal had to exhibit visible signs of illness, such as lethargy, coughing, or a runny nose, in addition to experiencing BW loss relative to its initial BW and having a rectal temperature exceeding 39.5°C. The treatment administration sequence involved a single subcutaneous injection of Draxxin (Tulathromycin, Pfizer, New York, NY), followed by an injection of Banamine (Flunixin meglumine, Merck Animal Health, Summit, NJ). If any animal did not respond to the initial treatment, second or third treatments were administered accordingly. At the conclusion of the 35-day period, the animals were categorized into two groups: (1) Animals diagnosed with bovine respiratory disease (BRD) at any time during the 35-day period (Sick group) and (2) Animals that did not exhibit any signs of BRD throughout the 35-day period (Healthy group). Prior to the morning feeding on day 35, blood samples (10 mL each) were collected from all beef steers. The blood was drawn from the coccygeal vessels using tubes containing sodium heparin (Fisher Scientific Company). Subsequently, plasma samples were prepared by centrifugation at 2,500 × g for 20 min at 4°C. The plasma samples were then stored at −80°C for subsequent analysis.

### Targeted metabolomics analysis using nuclear magnetic resonance spectroscopy

To evaluate the metabolic status of the two groups of beef steers, plasma samples collected on day 35 were subjected to metabolome analysis using Nuclear Magnetic Resonance (NMR) spectroscopy. A total of 50 metabolites, including organic acids, amino acids, hexoses, lipids, and carnitines were quantified using this technique (Supplementary Table S2). The plasma sample preparation and NMR spectral analysis procedures followed the protocols previously published by Ogunade et al. (21). Initially, a deproteinization step was conducted using ultra-filtration, following the method described by Psychogios et al. (22). This step aimed to eliminate macromolecules such as proteins and lipoproteins. Subsequently, 160 μL of the sample was combined with 40 μL of a standard buffer solution composed of 54% D2O and 46% 250 mM KH_2_PO_4_ at pH 7.0. The resulting plasma sample (200 μL) was then transferred into a 3 mm SampleJet NMR tube to undergo spectral analysis. All ^1^H-NMR spectra were acquired utilizing a 700 MHz Avance III spectrometer, which was equipped with a 5 mm HCN Z-gradient pulsed-field gradient cryoprobe. Bayesil, an automated analysis software package enabling qualitative and quantitative analysis (23), was used to process the ^1^H-NMR spectra. To minimize potential errors associated with compound identification and quantification, an additional inspection and verification process was conducted by an NMR spectroscopist.

### Statistical analysis

The performance and DMI results were analyzed using the GLIMMIX model of SAS (SAS 9.3, SAS Inst. Inc., Cary, NC) as a randomized block design, using steer as the experimental unit. Metabolome data was analyzed using Metaboanalyst 5.0 software (24). Prior to the statistical analysis, the data were log-transformed and auto-scaled. To identify the differentially abundant metabolites between the two groups of beef steers, a false discovery rate (FDR)-adjusted *p*-values threshold of ≤0.05 and an area under the curve (AUC) threshold of >0.70 were utilized. The receiver operating characteristic (ROC) curve analysis was performed by the ROCCET web server. To assess the metabolic pathways that were affected by BRD infection, a quantitative enrichment analysis of all the metabolites was conducted using the KEGG database.

## Results

At the end of the experiment, 31 beef steers were diagnosed with BRD at any time during the 35-day period (Sick group) and 46 beef steers did not exhibit any signs of BRD throughout the 35-day period (Healthy group). The results of the growth performance of the healthy and sick beef steers are shown in Table 1. Sick beef steers had lower (*p* = 0.01) ADG compared to healthy steers (1.44 vs. 1.64 kg/d). However, DMI was similar for the two groups. Consequently, the sick beef steers had greater (*p* = 0.01) feed:gain ratio compared to healthy ones (3.57 vs. 3.13).

**Table 1:** Performance of the sick and healthy beef steers during the 35-d receiving period.

	Sick	Healthy	SEM	*p*-value
ADG, kg/d	1.44	1.64	0.06	0.01
DMI, kg/d	5.13	5.27	0.15	0.33
Feed:gain	3.57	3.13	0.09	0.01

**Figure 1:**
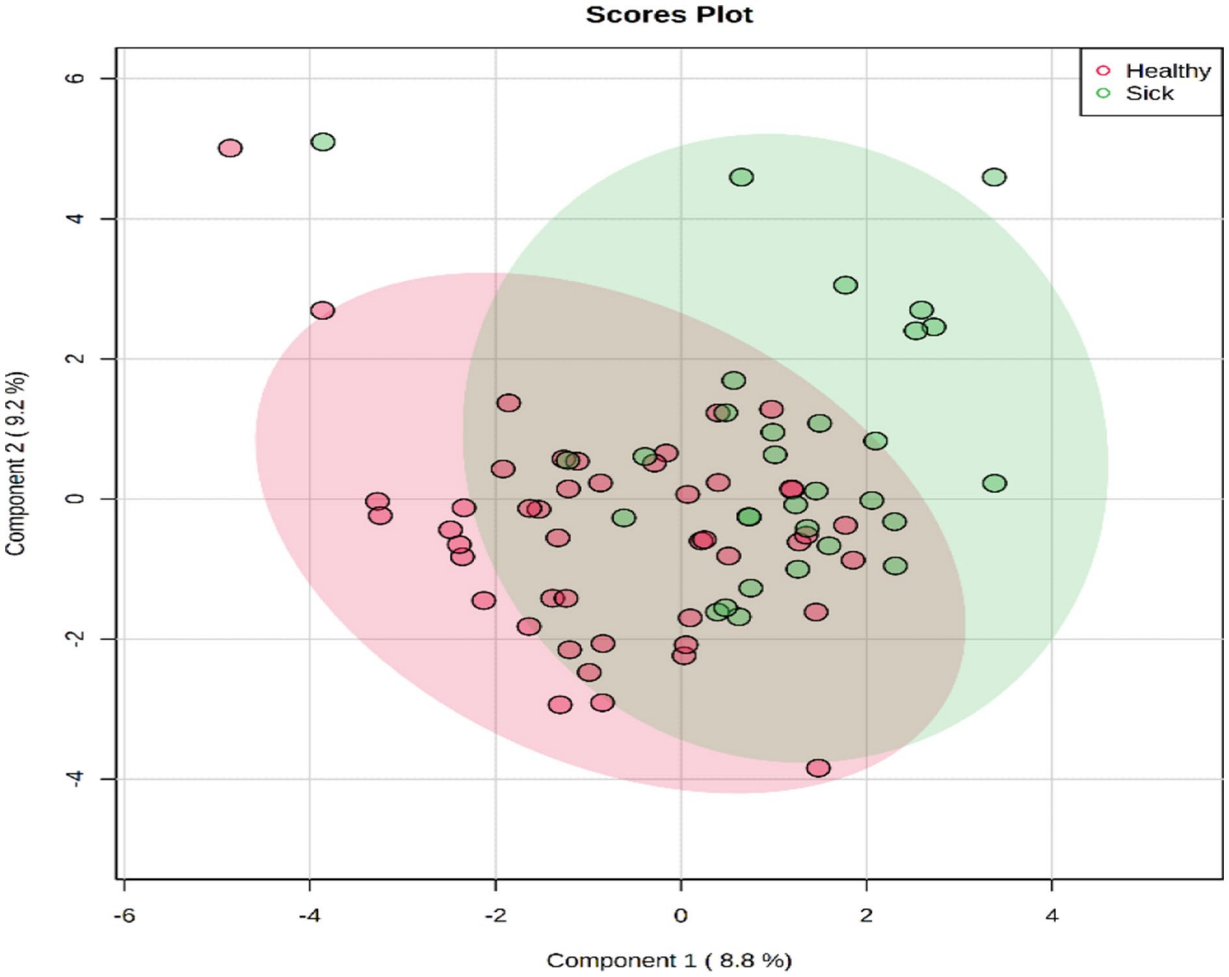
PLS-DA scores plot of plasma metabolome of Healthy and Sick beef steers.

**Figure 2:**
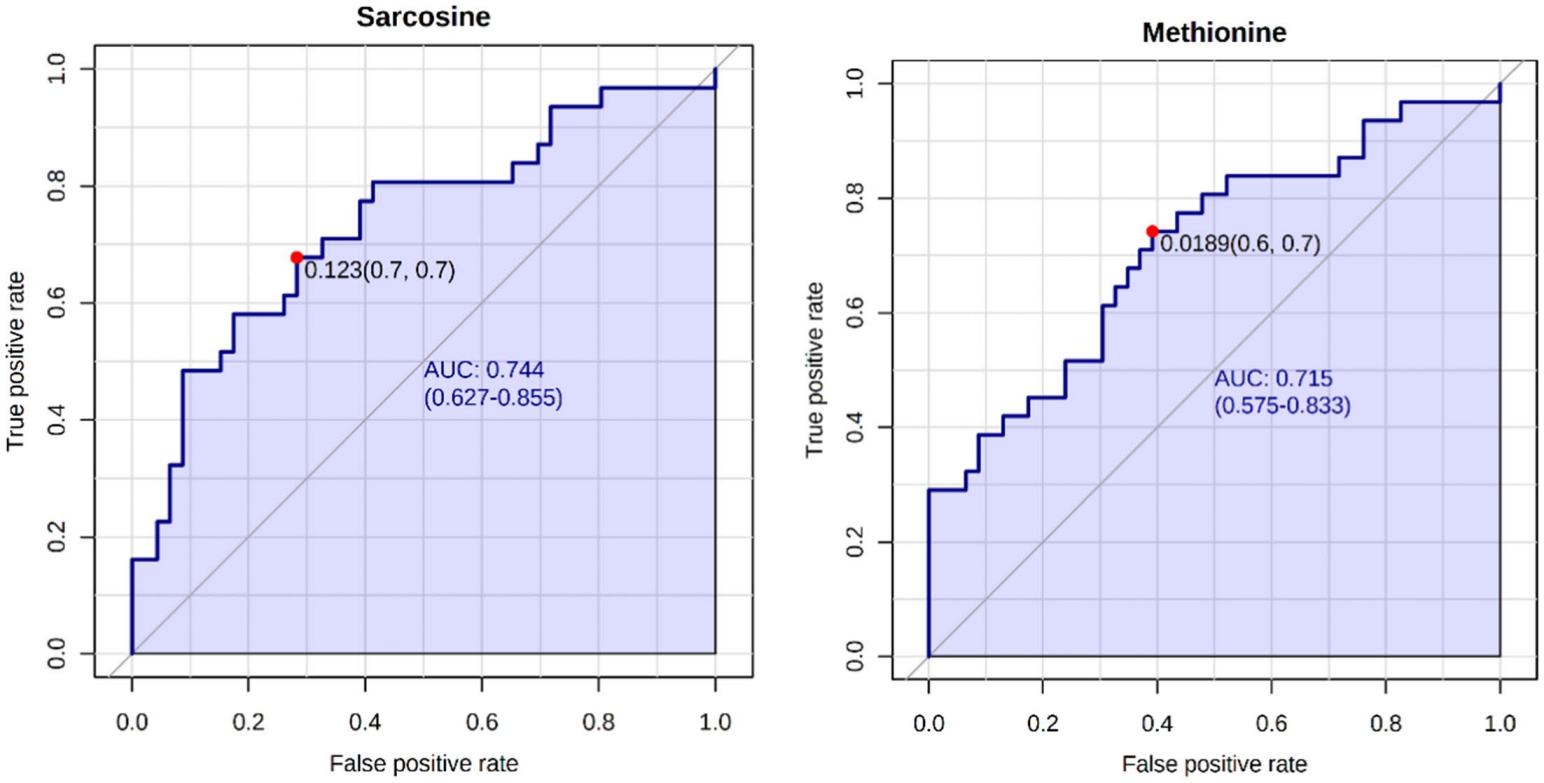
Biomarker analysis of plasma metabolome. ROC curve analysis of candidate plasma biomarkers (methionine and sarcosine) associated with BRD infection in the beef steers.

**Figure 3:**
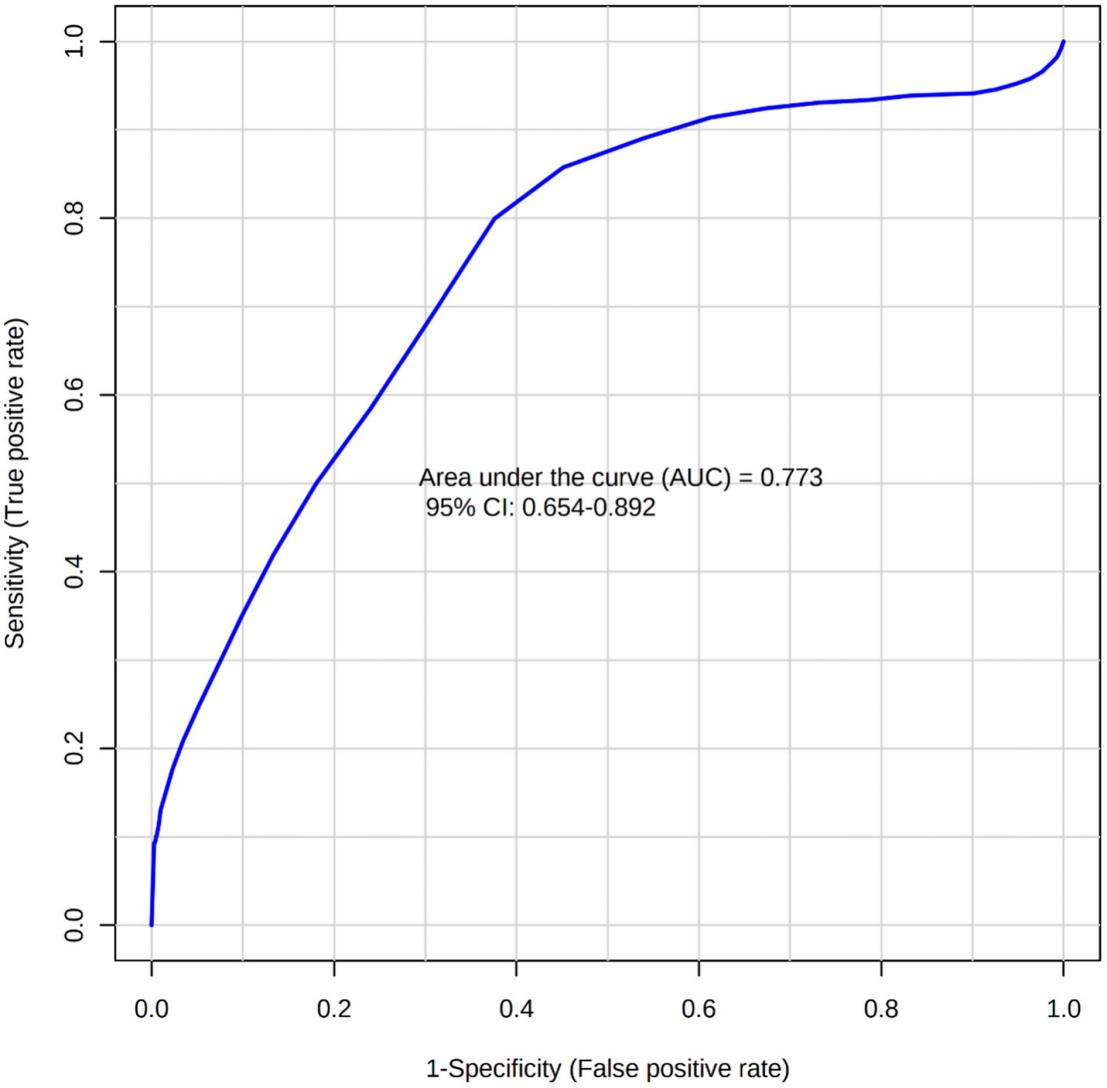
Biomarker analysis using multiple metabolites (Sarcosine and Methionine).

**Figure 4:**
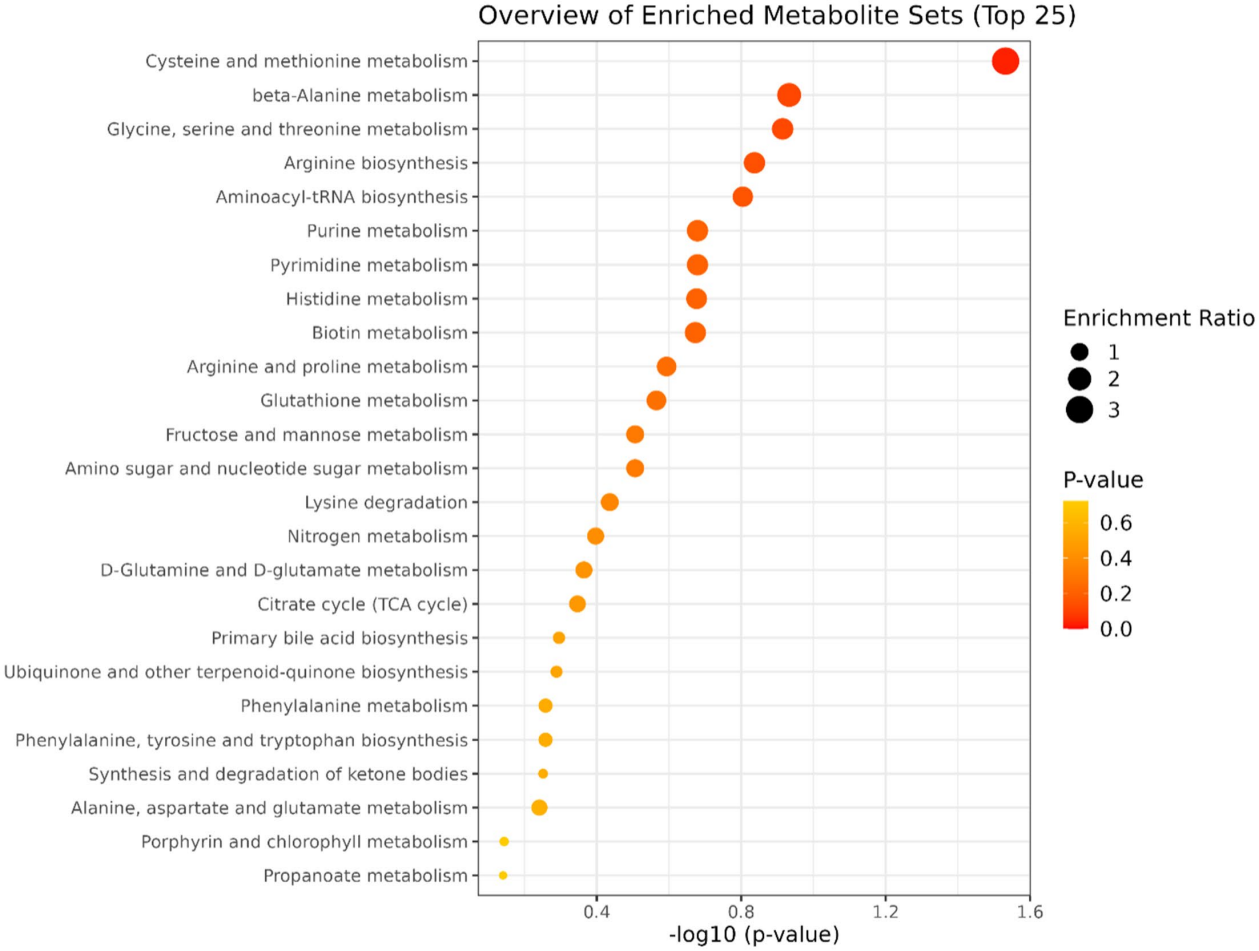
Results of the pathway enrichment analysis. Only cysteine and methionine metabolism had *p* ≤ 0.05.

A total of 50 metabolites were detected and quantified in the plasma samples of all the beef steers (Supplementary Table S2). The PLS-DA scores plot showed an overlap but with a slight separation of the entire plasma metabolome between the sick and healthy steers. Thus, suggesting that the plasma metabolome was altered by BRD infection (Figure 1). Plasma concentrations of four metabolites (sarcosine, methionine, dimethyl sulfone and L-histidine) were greater (FDR ≤ 0.05) in the healthy steers compared to the sick steers (Table 2). Out of the four differentially abundant metabolites, only two metabolites (sarcosine and methionine) had AUC values >0.70 (Figure 2). The results of the ROC analysis of the two metabolites revealed an AUC value of 0.773, suggesting that plasma concentrations of methionine and sarcosine could be used as a plasma biomarker panel associated with BRD infection in beef steers (Figure 3). The results of the quantitative enrichment analysis revealed cysteine and methionine metabolism (*p* = 0.05) was enriched in healthy, compared to the sick steers (Figure 4). The enriched metabolites associated with this pathway were methionine, cysteine, and pyruvic acid.

**Table 2:** Differentially abundant plasma metabolites in beef steers with or without bovine respiratory disease.

Metabolites	FC (healthy/sick)	FDR
Sarcosine	1.29	0.01
Methionine	1.13	0.01
Dimethyl sulfone	1.22	0.02
L-Histidine	1.06	0.04

Healthy = healthy beef steers, Sick = sick beef steers.

FC = fold change (healthy/sick).

Only metabolites with false discovery rate (FDR) ≤ 0.05 are shown.

## Discussion

The performance parameters of the sick and healthy steers during the receiving period were compared, revealing differences in ADG and feed:gain ratio. Sick steers exhibited lower ADG and higher feed:gain ratio compared to the Healthy steers, indicating that BRD may disrupt the metabolic processes involved in growth and nutrient utilization. Furthermore, though not statistically different, slightly lower DMI in sick steers suggests that BRD may affect satiety and feed intake. Similar effects of BRD on the performance of beef cattle have been reported in previous studies (25–27), suggesting that BRD negatively impacts nutrient absorption and utilization, alters metabolism, and increases energy expenditure due to the immune response and inflammation (28).

Bovine respiratory disease has a profound impact on the overall metabolic homeostasis, causing notable alterations in metabolite levels that reflect the body’s response to the infection and its defense mechanisms against the invading pathogens (29). The manifestation of BRD infection in beef cattle can have a significant impact on their metabolism, depending on the degree of pathological condition development (29). The disease disrupts various biological systems within the animal, particularly immune activation and nutrient metabolism (30, 31). The disruptions experienced as a result of these events lead to various biological alterations. One notable change is the upregulation of protein turnover, which is initiated by the production of cytokines in response to the pathogen. This heightened protein turnover necessitates an increased demand for amino acids to facilitate the synthesis of acute-phase proteins in the liver (29).

Metabolomics assessment revealed altered cysteine and methionine metabolism and identified specific plasma metabolites that differed between healthy and sick steers, including sarcosine, methionine, dimethyl sulfone, and L-histidine. These metabolites play crucial roles in biological and metabolic processes related to growth, production, and reproduction in farm animals (32). The decreased plasma concentration of sarcosine and methionine in sick steers suggests potential implications for the growth and energy substrate levels in the blood. Dysregulation in the metabolism of these amino acids may affect protein synthesis, muscle development, energy availability, and utilization, thereby impacting overall growth and body weight gain. Methionine, being an essential amino acid, is involved in various metabolic processes crucial for growth and muscle development. In the context of BRD, decreased methionine levels may compromise protein synthesis, utilization of other amino acids, dietary nutrient utilization, and immune defense mechanisms. Methionine deficiency has been associated with poor growth performance in growing beef cattle fed high forage diet such as the one fed in this study (33). Additionally, decreased methionine availability can compromise antioxidant defense mechanisms, leading to increased oxidative damage, inflammation, and impaired growth. Sarcosine is an intermediate product in the metabolism of glycine and methionine, thus, the reduced plasma concentration of sarcosine in sick beef steers may signify dysregulation in the metabolic pathways of these amino acids, which can have a significant impact on essential processes such as protein synthesis and muscle development which are critical for optimal growth in beef cattle. Furthermore, sarcosine is involved in the one-carbon metabolism pathway, which is crucial for the synthesis of important molecules such as DNA, RNA, and proteins. Disruptions in sarcosine and methionine metabolism can lead to reduced availability of metabolites involved in energy production, which may explain the reduced ADG of the sick steers observed in this study.

## Conclusion

Our findings demonstrate that BRD infection reduced the ADG and increased gain:feed ratio, and altered the plasma metabolome of the beef steers toward reduced concentrations of sarcosine, methionine, dimethyl sulfone and L-histidine. Additionally, quantitative enrichment analysis revealed that cysteine and methionine metabolism was enriched in healthy steers compared to sick steers. Further research is warranted to elucidate the underlying mechanisms and explore targeted interventions, such as nutritional supplementation of methionine, sarcosine, or cysteine, for mitigating the metabolic consequences of BRD in cattle.

## Data availability statement

The original contributions presented in the study are included in the article/Supplementary material, further inquiries can be directed to the corresponding author.

## Ethics statement

The animal study was reviewed and approved by West Virginia University (Protocol #2014-0194).

## Author contributions

IO designed the experiment. FE, GT, MI, IO, TS, ET, DO, and YL conducted the experiment and analyzed the data. FE drafted the manuscript. IO reviewed the final manuscript together with FE, GT, MI, IO, TS, ET, DO, and YL. All authors contributed to the article and approved the submitted version.

## Funding

This work was funded by West Virginia University Experimental Station in support of U.S. Department of Agriculture hatch multi-state regional project W-3010 Scientific Article Number 3463.

## Conflict of interest

The authors declare that the research was conducted in the absence of any commercial or financial relationships that could be construed as a potential conflict of interest.

## Publisher’s note

All claims expressed in this article are solely those of the authors and do not necessarily represent those of their affiliated organizations, or those of the publisher, the editors and the reviewers. Any product that may be evaluated in this article, or claim that may be made by its manufacturer, is not guaranteed or endorsed by the publisher.

## References

[1] 1Nicola I Cerutti F Grego E Bertone I Gianella P D'Angelo A. Characterization of the upper and lower respiratory tract microbiota in Piedmontese calves. Microbiome. (2017) 5:152. doi: 10.1186/s40168-017-0372-5 29157308 PMC5697440

[2] Short DM Lombard JE Earley B. The National Animal Health Monitoring System’s perspective on respiratory disease in dairy cattle. Anim Health Res Rev. (2020) 135–138. doi: 10.1017/S1466252320000080, PMID: 33682666

[3] Chai J Capik SF Kegley B Richeson JT Powell JG Zhao J. Bovine respiratory microbiota of feedlot cattle and its association with disease. Vet Res. (2022) 53:4. doi: 10.1186/s13567-021-01020-x, PMID: 35022062 PMC8756723

[4] Lynch E McGee M Earley B. Weaning management of beef calves with implications for animal health and welfare. J Appl Anim Res. (2019) 47:167–75. doi: 10.1080/09712119.2019.1594825, PMID: 27695797

[5] Earley B Buckham Sporer K Gupta S. Invited review: relationship between cattle transport, immunity and respiratory disease. Anim Int J Anim Biosci. (2017) 11:486–92. doi: 10.1017/S1751731116001622, PMID: 28209213

[6] Guzman E Taylor G. Immunology of bovine respiratory syncytial virus in calves. Mol Immunol. (2015) 66:48–56. doi: 10.1016/j.molimm.2014.12.004, PMID: 25553595

[7] El-Deeb WM Rizk MA Fayez MM Mkrtchyan HV Kandeel M. Clinical efficacy of antimicrobial agents in combination with flunixin meglumine and phenylbutazone on acute phase response in respiratory disease of calves. Pak Vet J. (2021) 41:71–7. doi: 10.29261/pakvetj/2021.001

[8] O’Connor A Hu D Totton S Scott N Winder C Wang B. A systematic review and network meta-analysis of bacterial and viral vaccines, administered at or near arrival at the feedlot, for control of bovine respiratory disease in beef cattle. Anim Health Res Rev. (2019) 20:143–62. doi: 10.1017/S1466252319000288 32081122

[9] O’Connor AM Hu D Totton SC Scott N Winder CB Wang B. A systematic review and network meta-analysis of injectable antibiotic options for the control of bovine respiratory disease in the first 45 days post arrival at the feedlot. Anim Health Res Rev. (2019) 20:163–81. doi: 10.1017/S1466252320000031 32081117

[10] Larson RL Step DL. Evidence-based effectiveness of vaccination against *Mannheimia haemolytica*, Pasteurella multocida, and *Histophilus somni* in feedlot cattle for mitigating the incidence and effect of bovine respiratory disease complex. Vet Clin N Am Food Anim Pract. (2012) 28:97–106, 106e1-7, ix. doi: 10.1016/j.cvfa.2011.12.005 22374120

[11] Theurer ME Larson RL White BJ. Systematic review and meta-analysis of the effectiveness of commercially available vaccines against bovine herpesvirus, bovine viral diarrhea virus, bovine respiratory syncytial virus, and parainfluenza type 3 virus for mitigation of bovine respiratory disease complex in cattle. J Am Vet Med Assoc. (2015) 246:126–42. doi: 10.2460/javma.246.1.126 25517335

[12] Marshall BM Levy SB. Food animals and antimicrobials: impacts on human health clin. Microbiol Rev. (2011) 24:718–33. doi: 10.1128/CMR.00002-11, PMID: 21976606 PMC3194830

[13] Garcia-Migura L Hendriksen RS Fraile L Aarestrup FM. Antimicrobial resistance of zoonotic and commensal bacteria in Europe: the missing link between consumption and resistance in veterinary medicine. Vet Microbiol. (2014) 170:1–9. doi: 10.1016/j.vetmic.2014.01.013, PMID: 24589430

[14] Murray GM O'Neill RG More SJ McElroy MC Earley B Cassidy JP. Evolving views on bovine respiratory disease: an appraisal of selected control measures - part 2. Vet J. (2016) 2016:78–82. doi: 10.1016/j.tvjl.2016.09.013 27810216

[15] Richeson JT Beck PA Gadberry MS Gunter SA Hess TW Hubbell DS III. Effects of on-arrival versus delayed modified live virus vaccination on health, performance, and serum infectious bovine rhinotracheitis titers of newly received beef calves. J Anim Sci. (2008) 2008:999–1005. doi: 10.2527/jas.2007-0593 18192559

[16] Blakebrough-Hall C Dona A D’occhio MJ McMeniman J González LA. Diagnosis of bovine respiratory disease in feedlot cattle using blood ^1^H NMR metabolomics. Sci Rep. (2020) 10:115. doi: 10.1038/s41598-019-56809-w 31924818 PMC6954258

[17] Johnson CH Ivanisevic J Siuzdak G. Metabolomics: beyond biomarkers and towards mechanisms. Nat Rev Mol Cell Biol. (2016) 17:451–9. doi: 10.1038/nrm.2016.25, PMID: 26979502 PMC5729912

[18] Wishart DS. Emerging applications of metabolomics in drug discovery and precision medicine. Nat Rev Drug Discov. (2016) 15:473–84. doi: 10.1038/nrd.2016.32, PMID: 26965202

[19] Santos-Rivera M Fitzkee NC Hill RA Baird RE Blair E Thoresen M. NMR-based metabolomics of plasma from dairy calves infected with two primary causal agents of bovine respiratory disease (BRD). Sci Rep. (2023) 13:2671. doi: 10.1038/s41598-023-29234-3 36792613 PMC9930073

[20] National Academies of Sciences, Engineering, and Medicine. Nutrient requirements of beef cattle: Eighth Revised Edition. Washington, DC: The National Academies Press (2016). 38386771

[21] Ogunade I Yun J James A Andre O Diwakar V Adegbola A. Biomarker of aflatoxin ingestion: 1H NMR-based plasma metabolomics of dairy cows fed aflatoxin B1 with or without sequestering agents. Toxins. (2018) 10, no. 12:545. doi: 10.3390/toxins10120545, PMID: 30567330 PMC6316819

[22] Psychogios N Hau DD Peng J Guo AC Mandal R Bouatra S. The human serum metabolome. PLoS One. (2011) 6:e16957. doi: 10.1371/journal.pone.0016957, PMID: 21359215 PMC3040193

[23] Ravanbakhsh S Liu P Bjordahl TC Mandal R Grant JR Wilson M. Accurate, fully-automated NMR spectral profiling for metabolomics. PLoS One. (2015) 10:e0124219. doi: 10.1371/journal.pone.0124219, PMID: 26017271 PMC4446368

[24] Pang G Shen C Cao L Hengel AVD. Deep learning for anomaly detection: a review. ACM Comput Surv. (2021) 54:1–38. doi: 10.1145/3439950

[25] Fulton RW Purdy CW Confer AW. Bovine respiratory disease research (1983-2009). Anim Health Res Rev. (2009) 10:131–9. doi: 10.1017/S146625230999017X, PMID: 20003649

[26] Holland BP Burciaga-Robles LO VanOverbeke DL Shook JN Step DL Richards CJ. Effect of bovine respiratory disease during preconditioning on subsequent feedlot performance, carcass characteristics, and beef attributes. J Anim Sci. (2010, 2010) 88:20190167:2486–99. doi: 10.2527/jas.2009-2428, PMID: 20190167

[27] Wilson BK Step DL Maxwell CL Gifford CA Richards CJ Krehbiel CR. Effect of bovine respiratory disease during the receiving period on steer finishing performance, efficiency, carcass characteristics, and lung scores. Prof Anim Sci. (2017) 33:24–36. doi: 10.15232/pas.2016-01554, PMID: 32288478 PMC7147665

[28] Henning AK Groschup MH Mettenleiter TC Karger A. Bovine respiratory disease complex in feedlot cattle: part 2. Risk factors for development, diagnosis, and persistence. Vet J. (2014) 199:175–80. doi: 10.1016/j.tvjl.2013.10.029, PMID: 24268478

[29] Krehbiel CR. Bovine respiratory disease influences on nutrition and nutrient metabolism In: Faculty papers and publications in animal science, vol. 1115 (2020) Available at: https://digitalcommons.unl.edu/animalscifacpub/1115 10.1016/j.cvfa.2020.03.010 32451030

[30] Baumann H Gauldie J. The acute phase response. Immunol Today. (1994) 15:74–80. doi: 10.1016/0167-5699(94)90137-6, PMID: 7512342

[31] Montgomery SP Sindt JJ Greenquist MA Miller WF Pike JN Loe ER. Plasma metabolites of receiving heifers and the relationship between apparent bovine respiratory disease, body weight gain, and carcass characteristics. J Anim Sci. (2009) 87:328–33. doi: 10.2527/jas.2008-0969, PMID: 18820162

[32] Zhao S Li H Han W Chan W Li L. Metabolomic coverage of chemical-group-submetabolome analysis: group classification and Four-Channel chemical isotope labeling LC-MS. Anal Chem. (2019) 91:12108–15. doi: 10.1021/acs.analchem.9b03431, PMID: 31441644

[33] Richardson CR Hatfield EE. The limiting amino acids in growing cattle. J Anim Sci. (1978) 46:740–5. doi: 10.2527/jas1978.463740x, PMID: 659344

