# Supplementary material for: Effects of bovine respiratory disease on the plasma metabolome of beef steers during the receiving period
Source: Front Vet Sci. 2023 Aug 4;10:1239651. doi: 10.3389/fvets.2023.1239651 (PMC10436613; doi:10.3389/fvets.2023.1239651)
Supplement: Supplementary file 1 [file Data_Sheet_1.DOCX]

Table S1. Ingredient and chemical composition of the basal diet

| Ingredient (%DM) | % of dietary DM |
| --- | --- |
| Corn silage | 64.0 |
| Mixed grass hay^1^ | 20.0 |
| Soybean meal | 8.12 |
| Dehydrated distillers’ grain | 2.58 |
| Soybean hulls | 4.07 |
| Limestone | 0.60 |
| Urea | 0.50 |
| Vitamin and mineral premix^2^ | 1.63 |
| **Nutrient analysis^3^** | |
| DM, % | 50.9 |
| CP, % | 15.1 |
| aNDF, % | 47.7 |
| ADF, % | 29.6 |
| TDN, % | 69.5 |
| NE_m_, Mcal/kg | 1.72 |
| NE_g_, Mcal/kg | 1.05 |

^1^Contains a mixture of orchard grass and fescue grass

^2^Guaranteed analysis: 15% Ca; 7.5% P; 20% salt; 1% Mg; 1% K; 3,600 mg/kg Mn; 12 mg/kg Co; 1,200 mg/kg Cu; 3,600 mg/kg Zn; 27 mg/kg Se; 60 mg/kg I; 660,000 IU/kg vitamin A; 660 IU/kg vitamin E; and 66,000 IU/kg vitamin D.

^3^DM = dry matter; CP = crude protein; aNDF = neutral detergent fiber (amylase treated); ADF = acid detergent fiber; EE = ether extract; TDN = total digestible nutrients; NE_m_ = net energy of maintenance; NE_g_ = net energy of gain.
